# Management of a Right Heart Intracavitary Thrombus in Transit in a Patient With Gastric Cancer in a Resource-Limited Setting: A Case Report

**DOI:** 10.7759/cureus.43133

**Published:** 2023-08-08

**Authors:** Marco Bermudez, Laura Pedraza, Nehemias Guevara, Gloria Erazo, Fernando R Valerio

**Affiliations:** 1 Internal Medicine, St. Barnabas Hospital (SBH) Health System, Bronx, USA; 2 Internal Medicine, Universidad Católica de Honduras, San Pedro Sula, HND; 3 Critical Care Medicine, Hospital Cemesa, San Pedro Sula, HND

**Keywords:** high-risk pulmonary embolism, thrombus in transit, gastric malignancy, surgical thrombectomy, cvc thrombolysis

## Abstract

A right atrial thrombus is an unusual source of imminent massive saddle pulmonary embolism (PE) . A hypercoagulable state secondary to gastric cancer (GC) can result in deep vein thrombosis (DVT) with a resultant right-sided heart thrombus in transit. Here, we present a case of a young male patient from Honduras with DVT and multiple venous thrombi extending from the external iliac veins to the suprahepatic left vein, inferior vena cava, and right atrium of the heart, secondary to a hypercoagulable state from GC, adenocarcinoma type. We describe the approach of treating a right heart intracavitary thrombus with imminent risk for saddle PE and sudden cardiac death with thrombolysis through a central venous catheter (CVC) in a resource-limited setting.

## Introduction

Gastric cancer (GC) is frequently complicated by hypercoagulation, with an associated risk of thromboembolic complications (TECs) [1]. An epidemiological study assessed the risk of having GC in Honduras, with a population-based cancer registry reporting an age-standardized risk of GC of 25.96 per 100,000 in males and 10.84 per 100,00 in females in a rural region representative of the population during five years of surveillance [2]. Treatment and histological classification also significantly influence the risk for TECs. Untreated gastric adenocarcinomas are associated with a higher risk for TECs [3]. The etiology of hypercoagulation in GC is multifactorial, including tumor-related prothrombotic mechanisms due to tumor cell-specific procoagulant properties and host cell inflammatory responses against the tumor [4]. This hypercoagulable state can predispose patients to thromboembolic events and sudden cardiac death. We present a case of a 41-year-old male patient with a past medical history of early-stage GC adenocarcinoma type, with a right heart intracavitary thrombus that failed treatment with thrombolytic therapy via a central venous catheter (CVC) and subsequent successful thrombus removal through salvage open heart surgery. There are no guidelines for treating a right atrial thrombus in transit or a consensus regarding the optimal treatment for its management. To our knowledge, this is the fourth case report in the medical literature describing CVC thrombolysis for a right heart atrial thrombus [5-7]. This case contributes to the medical literature on the management of patients with right heart thrombus in transit as it provides an example of a treatment outcome using CVC thrombolysis as a treatment option in a medical setting in which vacuum-assisted thrombectomy (VAT) is not immediately available for removal of a right atrial thrombus in transit with imminent risk for saddle pulmonary embolism (PE) and sudden cardiac death in a patient who is not a surgical candidate for open heart surgery and thrombectomy. This case describes a treatment outcome of CVC thrombolysis in a medical setting where VAT was not immediately available in a third-world country with limited resources. This case report was prepared following the CAse REport (CARE) guidelines [8].

This article was previously presented as a poster abstract at the 2023 American Thoracic Society Annual International Conference on May 22, 2023, in the Top Hematology/Oncology Critical Care case report category [9].

## Case presentation

A 41-year-old male with a past medical history of stage IB gastric adenocarcinoma in remission presented to the emergency department (ED) with a four-day history of fever, anorexia, night sweats, choluria, jaundice, right calf cramping pain, and a two-day history of dyspnea on exertion to moderate activity. The patient was diagnosed with gastric adenocarcinoma in the pylorus a year before admission, secondary to weight loss. The patient had a past medical history of gastritis of 20-year duration, unresponsive to over-the-counter proton pump inhibitors. During that period, the patient never sought formal medical evaluation. Endoscopy, gastric pyloric biopsy, and staging were consistent with gastric adenocarcinoma and *Helicobacter pylori* infection, during which he received *H. pylori *eradication treatment and underwent distal subtotal gastrectomy with regional lymphadenectomy eight months prior to admission. Staging and pathology findings were consistent with stage IB gastric adenocarcinoma. Postoperative clinical management and surveillance were suboptimal as the patient was lost to follow-up.

Vital signs in the ED showed blood pressure of 140/99 mmHg, heart rate (HR) of 128 per minute, respiratory rate (RR) of 24 per minute, and a fever of 38.4°C. Physical examination was remarkable for jaundice, no palpable lymphadenopathy, a protuberant abdomen tympanic to percussion, a painful mass in the epigastrium, and right leg pitting edema under the right knee, with redness, warmth, and red darkened skin. Laboratory results were significant for leukocytosis of 14.700 mm^3^, neutrophilia of 89.9% (absolute count of 13,200), thrombocytopenia of 60.000 mm^3^, alpha-fetoprotein of 0.94, and cancer antigen 19-9 (CA 19-9) of 237.5 (Table 1).

**Table 1:** Initial diagnostic workup of the patient and its trend during hospitalization and follow-up. WBC: white blood cell; BUN: blood urea nitrogen; PCO_2_:_ _partial pressure of carbon dioxide; PO_2_: partial pressure of oxygen; HCO_3_: bicarbonate; PT: prothrombin time; PTT: partial thromboplastin time; INR: international normalized ratio; CA 19-9: cancer antigen 19-9

	Day 1	Day 2	Day 3	Day 4	Day 5	Day 6	Day 7	Day 8	Day 9	Day 10	Day 11	Day 12
Ferritin (12-300 ng/mL)	1.183		2.613									
WBC (4.50-11.00 x10^3^/uL)	14.7	12.2	19.7	18.4		34	24.6	30	34.4	30.7	25.8	18.7
Hemoglobin (12.0-15.7 g/dL)	14.7	13.5	11.8	10.4		8.74	8.5	11.9	11.1	10.2	10.3	9.1
Platelet (140-440x10^3^/uL)	60	40	55	154		207	254	299	320	376	478	430
Creatinine (0.50-1.50 mg/dL)	1.1	1.42	0.89	0.6	0.5	0.7	0.8	0.8	0.7	0.6	0.72	0.7
BUN (9-27 mg/dL)	27.1	36.6	28.8	17.4	11.1		24	29.1	21.8	16	17.6	16.9
Urea (6-24 mg/dL)	58	78	62	37	24		52	62	47	34	38	36
Albumin (3.8-4.9 d/dL)								3.1				
C-reactive protein (0.0-0.9 mg/dL)												
Procalcitonin (<0.1 ng/mL)	>100	>100	89.8		32.81	37.64	31.82	16.49	0.31	6.52	8.17	12.24
Total bilirrubin (0.1-1.2 mg/dL)	4.6		4.5	4.7	4.1				2.3	1.4		
Direct bilirrubin (<0.3 mg/dL)	3.5		4.1	4.4	4				2.1	1.6		
Indirect bilirrubin (0.2-0.8 mg/dL)	1.1			0.3	0.1				0.2	-0.2		
Lactate dehydrogenase (105-333 IU/L)	326											
Alkaline phosphatase (44-147 IU/L)	603					891						
pH (7.35-7.45)	7.49	7.41				7.4	7.31	7.48	7.41	7.46	7.45	7.33
PCO_2_ (35-45 mmHg)	27.8	37.8				35.5	48.2	34.8	34.4	39	42	56.4
PO_2_ (75-100 mmHg)	61	44				125	48	114	90	96	105	54
HCO_3_ (22-26 mEq/L)	21.4	24				23.3	24.5	26.4	22	28	29	30.1
Base excess (-2 to +2 mEq/L)	-2	0				-1	-2	3	-3		5	4
Calcium (8.6-10.3 mg/dL)		9.9			8.5	8	8.8	9				
Chloride (96-106 mEq/L)		97.7					96.3	101	102	102	99.5	102.7
Magnesium (1.3-2.1 mEq/L)		2	1.6	2	2	3	2.2		1.6	1.8	1.9	2.2
Potassium (3.6-5.2 mmol/L)		3.06	3.6	3.85	4.68	4.8	4.34		3.7	3.9	4.74	4.85
Sodium (135-145 mEq/L)		129	132	130	131	134	135	135			136	136.3
D-dimer (<0.50)			3.9			3.96			8.05	>10		>10
PT (11-13.5 seg)			13.9	13			12.2	16.1			15.3	
PTT (25-35 seg)			24.2	23.8			21	26.7			33.5	39
INR (<1.2)			1	1			1	1			1	
CA 19-9 (0.0-37.00 U/ml)	237.5											
Alpha-fetoprotein (0.89-8.98 ng/ml)	0.94											

**Figure 1:**
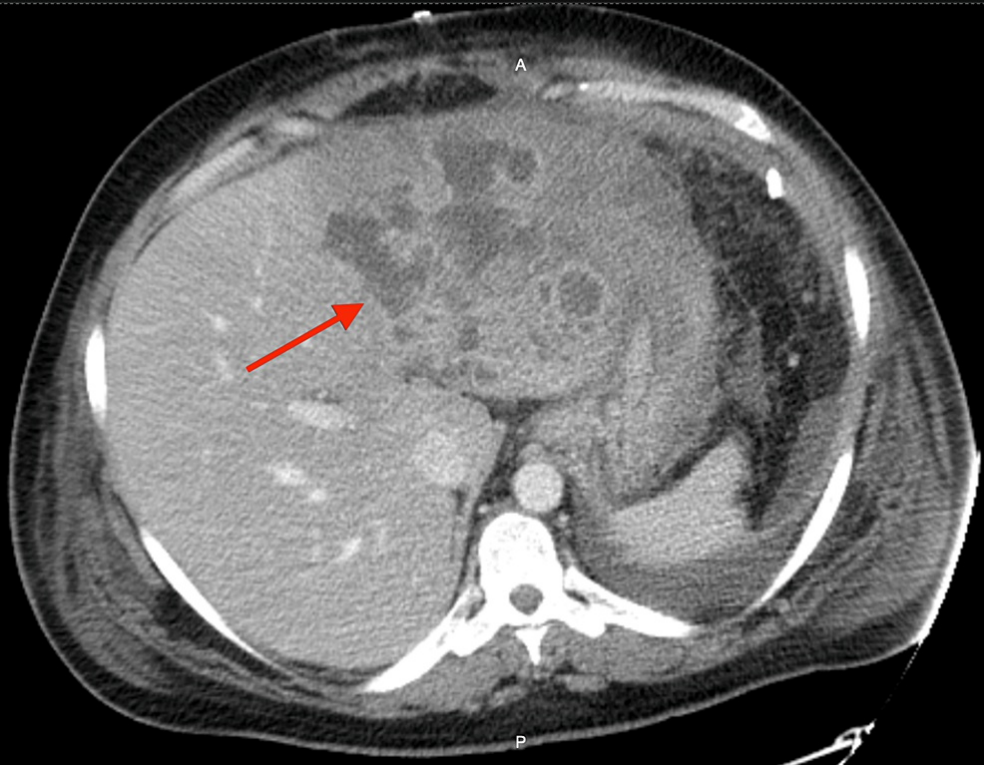
Computer tomography of the abdomen with arrows pointing to multilocular type lesions with areas of gas and necrosis consistent with hepatic abscess vs. hepatic metastasis from previously resected gastric cancer.

**Figure 2:**
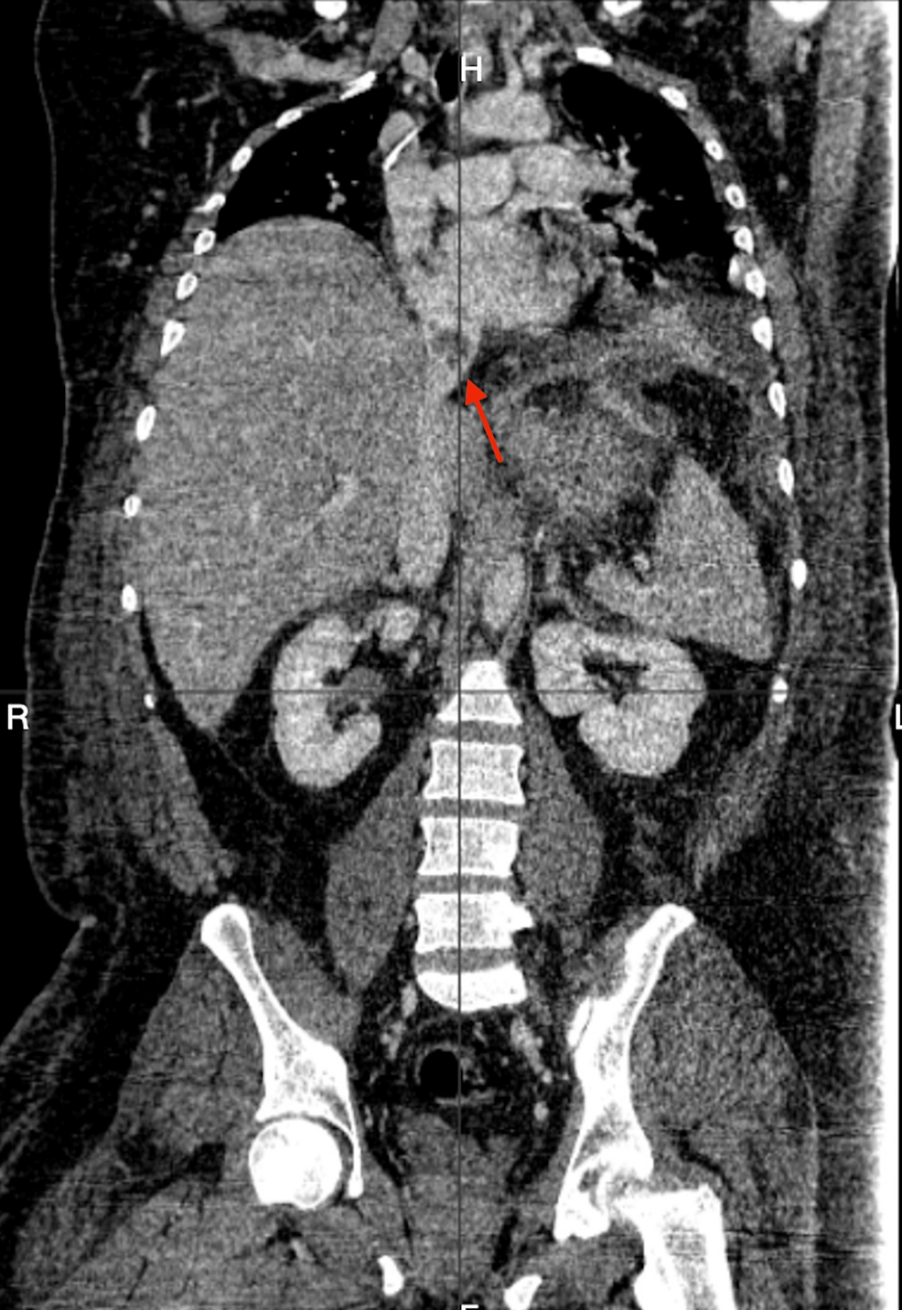
Computer tomography angiography showing thrombosis of the inferior vena cava.

Ultrasound of the deep veins demonstrated signs of deep vein thrombosis (DVT) of the right lower extremity. A computed tomography (CT) angiography of the chest and abdomen was performed to rule out PE and any intra-abdominal pathology, especially liver metastasis, as the patient had a history of GC and a clinical picture of hepatic/biliary pathology. CT angiography of the chest did not demonstrate a PE. CT angiography of the abdomen demonstrated thrombosis of the inferior vena cava (IVC) with extension to the suprahepatic left vein. It also demonstrated a hepatic mass interfering with the minor gastric curve and the posterior inferior left lobe of the liver, suggesting metastatic disease versus hepatic abscess (Figures 1, 2). Ultrasound of the liver was remarkable for a liver mass suspicious of malignancy with no findings of biliary pathology. Consequently, our patient received treatment at admission with subcutaneous enoxaparin 80 mg every 12 hours, intravenous (IV) meropenem 2 g every eight hours, IV metronidazole 500 mg every eight hours, IV vancomycin 1 g every 12 hours and IV maintenance fluids.

CT venography demonstrated thrombosis of the external iliac veins, abdominal vena cava (AVC), and IVC. Enoxaparin was held. Interventional radiology performed an image-guided biopsy of the hepatic lesion. Biopsy yielded a purulent fluid sent to the laboratory for cultures and cytology, confirming the diagnosis of a hepatic abscess without evidence of a metastatic disease. A bacterial culture of the hepatic abscess grew extended-spectrum beta-lactamase *Morganella morganii*. Enoxaparin was held as the patient developed hemorrhagic ascites post drainage of the hepatic abscess, in which the patient was stabilized with IV fluids. After stabilization and no more evidence of hemorrhage, enoxaparin was restarted. The same antibiotic regimen was continued.

On hospital stay day 3, the patient continued to be tachycardic, febrile, and dyspneic with the emergence of hypoxemia, with an oxygen saturation of 89% on 6 L per minute O_2_ supplementation through a nasal cannula. As there was a concern for endocarditis and septic emboli, a transthoracic echocardiogram was performed, which was unremarkable for endocarditis. Consequently, transesophageal echocardiography (TEE) was performed, which revealed a mobile round mass of 2.55 mm x 1.5 mm near the origin of the IVC to the right atrium with a hyperdynamic mobile, filiform mass near the drainage of the atrium (Video 1). Due to the thrombus extension, an IVC filter was not possible, and minimally invasive peripheral percutaneous VAT was not offered due to the lack of immediate availability in the hospital. Right atrial thrombectomy through an open cardiac surgery approach was also not possible as the patient was a poor surgical candidate due to an insurmountable perioperative risk of death. As the intracardiac mass had an imminent risk for dislodgement and consequential massive saddle PE with a substantial risk for sudden cardiac death, cardiothoracic surgery recommended localized CVC thrombolysis for the intracardiac mass with alteplase infusion as there was high suspicion for an intracardiac thrombus in transit. 

**Video 1:** TEE bicaval view showing a round mass of 2.5mm x 1.5 mm near the connection between the vena cava and right auricle, with highly mobile filiform extension near the drainage of the vena cava to the right auricle. TEE: transesophageal echocardiography

After a discussion with the patient and family members regarding the risk and benefits of CVC line insertion and CVC-guided thrombolytic therapy, a CVC was inserted with confirmation of cavoatrial placement with a chest X-ray. CVC thrombolysis was performed with an infusion of alteplase 50 mg in 200 ml of IV 0.9% sodium chloride via a right internal jugular vein (RIJ) CVC at a rate of 2 mg/hour to complete 25 hours of infusion. During the infusion, the patient went into cardiac arrest due to the suspected dislodgement of a piece of the intracardiac mass. The thrombolysis infusion was stopped, and cardiopulmonary resuscitation was immediately started. The patient was intubated, and after three minutes, the patient achieved a return of spontaneous circulation. Thrombolysis infusion was resumed and was completed with no further complications. Repeat TEE revealed a slight decrease in the size of the right intracavitary mass, with hyperdynamic mobility, suggesting imminent saddle PE. Cardiothoracic surgery was consulted for consideration of open heart surgery after risk and benefits were discussed with surrogate decision makers of the patient who agreed to the procedure.

**Figure 3:**
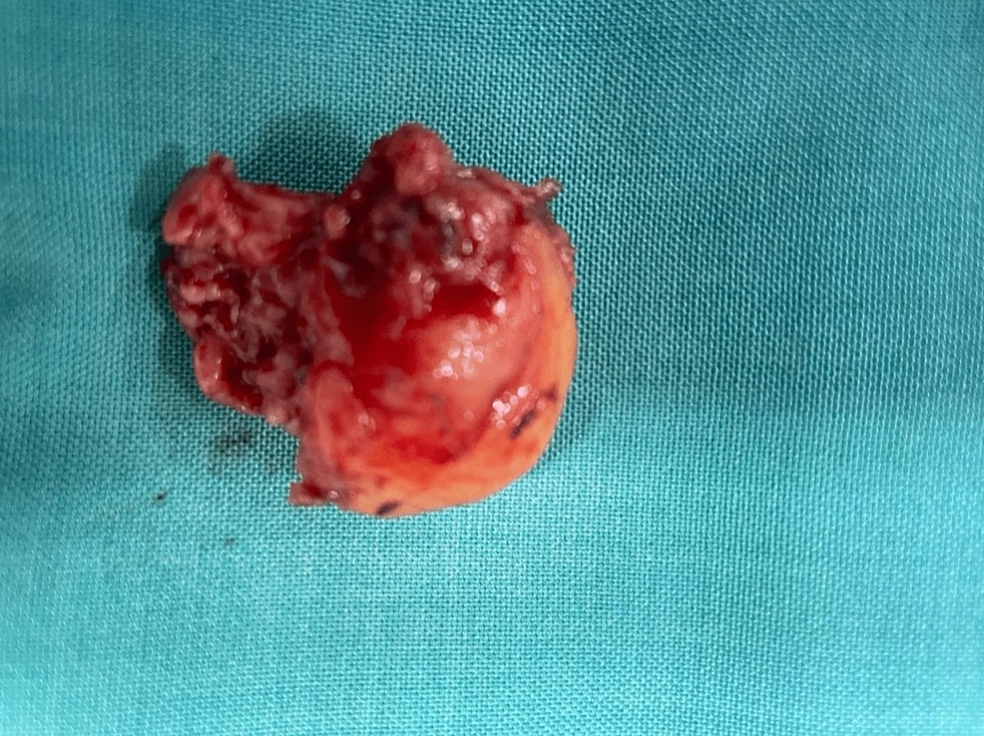
The gross specimen of the right atrial thrombus, measuring 0.3 cm x 0.3 cm, taken from the right auricle thrombectomy through an open heart surgery.

**Figure 4:**
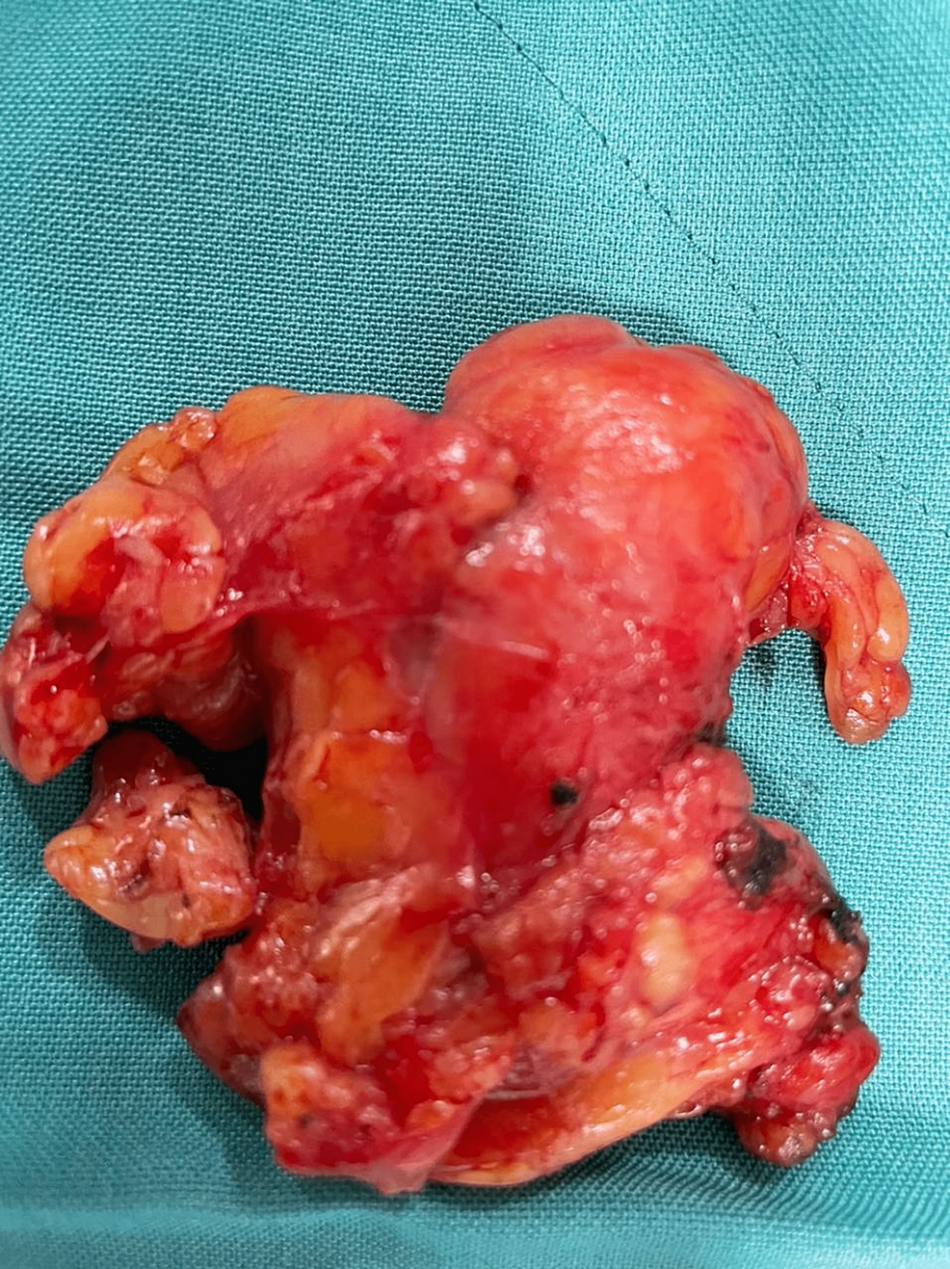
Gross thrombus specimen taken from thrombectomy of the inferior vena cava through an open heart surgery.

During the open heart surgery under cardiopulmonary bypass, the right atrial mass (Figure 3), thrombus in the IVC (Figure 4), thoracic lymphatic nodes, and pericardial nodes were removed and sent for pathology consultation. The patient clinically improved after surgery, with a resolution of sepsis, and was successfully extubated. Biopsy results reported thymus residues, lymphatic nodes, pericardial nodules, and an intracardiac mass consistent with a thrombus, all masses with no evidence of GC metastasis. A liver biopsy was also negative for malignancy and was consistent with a hepatic abscess. The patient was stabilized and discharged a week after the open heart surgery. The patient was discharged with apixaban 10 mg twice a day for one week and, after one week, 5 mg twice a day for venous thromboembolism (VTE) prophylaxis for an indefinite time and oral levofloxacin 750 mg once a day for seven days with a follow-up in one week, in which the patient reported resolution of respiratory symptoms, resolution of DVT symptoms in the right leg, lack of postoperative fever, and no signs of infection of the operative site.

## Discussion

Hypercoagulability state secondary to GC is a well-known condition. Our case is particular due to a massive PE caused by a right heart intracardiac mass, most likely secondary to a hypercoagulable state produced by a previously resected early-stage gastric adenocarcinoma. Our patient shared risk factors associated with thrombotic events in GC. A study described an increased risk of thrombotic events in patients in males with GC, recent surgery, and adenocarcinoma histology [3]. Moreover, thrombotic events in patients with GC are more common in patients with pyloric GC [3]. 

GC can also frequently cause VTE and PE in the postoperative setting of GC resection [10]. A retrospective study studying the incidence of VTE and PE in patients with a history of GC adenocarcinoma confirmed by pathology with age greater than 18 years old demonstrated that the majority of VTE events happened in patients with active disease and in the ambulatory setting, defined as after four weeks of most recent hospitalization, in which the majority of VTE and PE events happened in the setting of no chemoprophylaxis given for VTE during this period [10]. Moreover, VTE was reported in 12% of patients reported to be disease-free after a 12-month surveillance period after GC surgical resection [10]. A proportion of patients (23%) with a history of GC adenocarcinoma had the lower extremities as the site of VTE, and 38% had other sites of VTE that did not include the upper extremity, lower extremity, and PE [10]. In addition, 28% of patients with a history of GC had PE as their site of VTE [10]. Our patient was considered to be GC disease-free and was in the ambulatory postoperative setting of partial gastrectomy, not taking chemoprophylaxis for VTE, demonstrating that the hypercoagulable-state GC adenocarcinomas can produce even if patients are considered disease-free, which could result in multiple sites of VTE and ultimately a PE event [10]. 

Intracardiac masses can also be a frequent cause of pathology in the hospital. The most frequent etiology of intracardiac masses is thrombi, with an incidence of 53.54% among patients with intracardiac masses [11]. The second most common cause of intracardiac masses is tumors, most commonly benign, an example being myxomas [11]. Metastatic disease has also been reported as an origin for intracardiac masses [11]. The most frequently seen are metastatic lymphomas, in which B-cell lymphoma is the predominant type [11]. Most intracardiac thrombi with non-cardiac disorders are associated with malignancies and are commonly located in the right atrium [11]. Our patient was found to have an intracardiac right atrium thrombus that was not associated with a cardiac disorder. It was initially thought that it could have been related to the metastatic tissue of the GC; however, there was no metastatic tissue in the final pathology report. Current literature on GC has also shown that the incidence of metastatic disease is higher in men than in women. The liver has been described as the most frequent site of metastasis of GC [12]. Our patient followed the epidemiological trend; however, our patient did not have a metastatic mass but rather a liver abscess confirmed by pathology through interventional radiology-guided aspiration and biopsy results.

Multiple mechanisms lead to hypercoagulability in our patient. One is the mechanical compression produced by the hepatic abscess to the veins and consequential disturbed laminar blood flow. The second was the ability of GC to produce procoagulant factors, such as the tissue factor, which is released persistently in the presence of pro-inflammatory stimuli, such as tumor necrosis factor-alpha, interleukin-1B, and bacterial lipopolysaccharides [4]. A hypercoagulable-state post resection of intra-abdominal malignancies has also been well documented, with a persistence of a hypercoagulable state one-month post resection in 1/3 of cancer patients [13]. We want to add that a limitation of our case was that no workup was done to rule out secondary hypercoagulation disorders.

Associated right auricle thrombi with IVC thrombi incidence in the setting of malignancy has also been described in the literature. An autopsy study done in 2011 evaluated the origin of intracardiac thrombi from non-cardiac causes. The authors analyzed 11,724 autopsies performed in 15 years, in which 276 patients had intracardiac thrombi [14]. Of those patients, 16% of patients had intracardiac thrombi unrelated to primary cardiac diseases, and from that same group, 24% had intracardiac thrombi related to cancers [14]. The right atrium thrombi were related to IVC thrombosis in two out of 11 patients with intracardiac thrombi related to malignancy [14]. Our patient had a right atrium thrombi associated with IVC thrombosis related to GC malignancy. 

Gastrointestinal cancers have also been reported with the highest risk of DVT/PE among malignancies demonstrated by a study based on Medicare data, with stomach cancer ranking five out of 18 malignancies, with a rate of 85 DVT/PE events per 10,000 patients [15]. Other studies have found a higher incidence of VTE in patients hospitalized with malignancies, in which GC had an RR of 2.9 for VTE in patients hospitalized with malignancy aged 40-59 years old, only overtaken by cancers with origin in the pancreas, brain, and myeloproliferative cancer disorders [16]. 

Several case reports of CVC-directed thrombolysis for right atrial thrombus have been reported in the literature [5-7]. Most cases have been right atrial thrombi produced by complications of thrombosis of CVC, hemodialysis catheters, and intracardiac defibrillators, but few have reported malignancy-associated right atrial and IVC thrombi. Our case is unique as the massive extension of multiple thrombi from the external iliac veins, suprahepatic left vein, and IVC can only be explained by the blood stasis and inflammation produced by the hepatic abscess and hypercoagulability state produced by the gastric adenocarcinoma in our patient. A case report described a successful outcome using CVC thrombolysis for a right atrial thrombus caused by an intracardiac defibrillator for a patient who was a poor candidate for surgical resection or catheter embolectomy [7]. Our patient failed this approach, and a right atrial thrombectomy through an open heart surgery was needed to remove the mass and prevent further PEs, potential saddle PE, and sudden cardiac arrest. Prior reports have suggested mixed results with systemic anticoagulation and right atrial thrombectomy. Prior studies analyzing right atrial CVC thrombolysis for right atrial thrombus masses related to thrombosis of hemodialysis catheters also have demonstrated poor resolution results [17]. However, it is still considered an option for right atrial thrombus treatment caused by hemodialysis catheters in a meta-analysis study [17]. 

A thrombus in transit in the right heart with imminent risk for massive PE has up to a 90.9% mortality rate if left untreated [18]. Its most optimal treatment is still widely debated, mainly depending on the severity of the coexisting PE, the clinical state of the patient (which may contraindicate surgical embolectomy), comorbidities, contraindications, and technical resources. The most up-to-date treatment in a resource-rich setting for an intra-cardiac thrombus in transit with contraindications for thrombolysis or surgical embolectomy is mechanical endovascular thrombectomy through peripheral percutaneous-assisted thrombectomy [19]. Unfortunately, large prospective and retrospective studies are lacking in reporting its effectiveness compared to other treatment modalities. Its effectiveness has been reported primarily through the collection of case reports reporting its usage, in which one recent review article reported an up to 83.3% success rate in removing iliocaval and right intracardiac thrombi [19].

## Conclusions

To our knowledge, this is the first case report describing a failed right atrial thrombolysis through a CVC for a right heart intracardiac thrombus resulting from a hypercoagulable state from early-stage previously resected GC. This case illustrates the potential for life-threatening VTE events in the postoperative period of GC. VTE prophylaxis should be considered to prevent such events. This case also illustrates the life-threatening nature of a thrombus in transit and the need for prompt treatments. VAT is the least invasive and most effective of the currently available treatment options. Unfortunately, it is a procedure not immediately available in third-world countries. Although thrombolysis administered via a CVC is an infrequent treatment option, it can be considered in a resource-limited setting. Its failure may require salvage surgery through an emergent open heart surgery and thrombectomy for an intracardiac thrombus in transit with potentially life-threatening PE.
